# Development of an orally delivered GLP-1 receptor agonist through peptide engineering and drug delivery to treat chronic disease

**DOI:** 10.1038/s41598-021-01750-0

**Published:** 2021-11-18

**Authors:** Sergei Pechenov, Jefferson Revell, Sarah Will, Jacqueline Naylor, Puneet Tyagi, Chandresh Patel, Lihuan Liang, Leo Tseng, Yue Huang, Anton I. Rosenbaum, Kemal Balic, Anish Konkar, Joseph Grimsby, J. Anand Subramony

**Affiliations:** 1 Drug Delivery, Dosage Form Design and Development, AstraZeneca, Gaithersburg, MD USA; 2 Discovery Sciences, AstraZeneca R&D, Cambridge, UK; 3 Bioscience Metabolism, Research and Early Development, Cardiovascular, Renal and Metabolism (CVRM), Biopharmaceuticals R&D, AstraZeneca, Gaithersburg, MD USA; 4 Bioscience Metabolism, Research and Early Development, Cardiovascular, Renal and Metabolism, BioPharmaceuticals R&D, AstraZeneca, Gothenburg, Sweden; 5 Bioscience Renal, Research and Early Development, Cardiovascular, Renal and Metabolism (CVRM), BioPharmaceuticals R&D, AstraZeneca, Cambridge, UK; 6 Bioscience Renal, Research and Early Development, Cardiovascular, Renal and Metabolism (CVRM), BioPharmaceuticals R&D, AstraZeneca, South San Francisco, CA USA; 7 Integrated Bioanalysis, Clinical Pharmacology and Quantitative Pharmacology, Clinical Pharmacology & Safety Sciences, R&D, AstraZeneca, South San Francisco, CA USA; 8 Biologics Engineering, R&D, AstraZeneca, Gaithersburg, MD USA

**Keywords:** Diabetes, Type 2 diabetes, Drug discovery, Drug delivery

## Abstract

Peptide therapeutics are increasingly used in the treatment of disease, but their administration by injection reduces patient compliance and convenience, especially for chronic diseases. Thus, oral administration of a peptide therapeutic represents a significant advance in medicine, but is challenged by gastrointestinal instability and ineffective uptake into the circulation. Here, we have used glucagon-like peptide-1 (GLP-1) as a model peptide therapeutic for treating obesity-linked type 2 diabetes, a common chronic disease. We describe a comprehensive multidisciplinary approach leading to the development of MEDI7219, a GLP-1 receptor agonist (GLP-1RA) specifically engineered for oral delivery. Sites of protease/peptidase vulnerabilities in GLP-1 were removed by amino acid substitution and the peptide backbone was bis-lipidated to promote MEDI7219 reversible plasma protein binding without affecting potency. A combination of sodium chenodeoxycholate and propyl gallate was used to enhance bioavailability of MEDI7219 at the site of maximal gastrointestinal absorption, targeted by enteric-coated tablets. This synergistic approach resulted in MEDI7219 bioavailability of ~ 6% in dogs receiving oral tablets. In a dog model of obesity and insulin resistance, MEDI7219 oral tablets significantly decreased food intake, body weight and glucose excursions, validating the approach. This novel approach to the development of MEDI7219 provides a template for the development of other oral peptide therapeutics.

Peptide therapeutics have become increasingly common in the treatment of diseases, both acute and chronic^1^. However, their remarkable potential is not without obstacles, one of which is the mode of administration by subcutaneous injection. Oral administration offers greater convenience for patients who require long-term treatment and is more appealing for those with a fear of injections^2–5^. Furthermore, oral administration may improve efficacy through improved compliance and earlier initiation of treatment compared with injectables^6,7^. Peptide therapeutics are not naturally suited for oral administration because of their inherently low gastrointestinal permeability, high susceptibility to degradation by gastrointestinal and serum proteases, and rapid renal clearance^8^. Unless stabilized and formulated to overcome these obstacles, orally delivered peptides suffer from poor bioavailability and short half-lives, which may limit efficacy even with frequent dosing. Overcoming these challenges represents an important research goal in the development of new peptide therapeutics for metabolic and other chronic diseases. Several approaches have been tested in preclinical and clinical studies to enhance the transport of peptides across the gastrointestinal tract wall^9^. Administration of peptides with permeation enhancers, which transiently alter epithelial tight junctions to increase permeability, has been the most studied approach. Oral formulations of calcitonin^10^, octreotide^11^ and insulin^12^ with permeation enhancers have all been tested in clinical studies up to phase 3 and detailed pharmacokinetics have been established. An alternative approach is the use of nano- and microsystems, for preferential uptake up by Peyer’s patches in the gastrointestinal tract and transport via the lymphatic system^13^. Inorganic (e.g. silica^14^) and organic (e.g. lipid^15^) matrices have been explored for delivery of insulin, octreotide and other peptides via Peyer’s patches. Nano- and microsystems can also enhance the efficacy of permeation enhancers by colocalization with peptides^16^. More recently, disruptive technologies such as coated or integrated drug microneedles containing peptide have been advanced into the clinic (e.g. RaniPill containing octreotide^17^, NCT03798912). High-pressure liquid jets of peptide formulations have also been proposed as a methodology for non-invasive oral peptide delivery to enhance bioavailability^18,19^.

Here we have used glucagon-like peptide 1 (GLP-1) as a ‘model’ injected peptide therapeutic used to treat obesity-linked type 2 diabetes, a very common chronic disease. We describe a comprehensive multidisciplinary approach for the development and optimization of a new GLP-1 receptor agonist (GLP-1 RA), MEDI7219, specifically engineered for digestion resistance and formulated for oral delivery.

GLP-1 RAs are established therapeutics shown to provide clinically meaningful reductions in elevated blood sugar, glycated hemoglobin (HbA_1C_), body weight, blood pressure and cardiovascular risk in patients with type 2 diabetes and obesity^20–23^. All of the six GLP-1RAs approved by the US Food and Drug Administration require subcutaneous administration, with the exception of an oral formulation of semaglutide^22,24,25^.

GLP-1 is a 30-amino acid incretin peptide hormone produced by cleavage of proglucagon, and is secreted mainly by the enteroendocrine L-cells of the distal ileum in response to nutrient ingestion^21,26^. By activating its cognate G protein-coupled receptor (GLP-1R) primarily on pancreatic β-cells, GLP-1 stimulates glucose-dependent insulin release, leading to glucose uptake and improved glycemic control^26,27^. Furthermore, GLP-1 suppresses pancreatic glucagon secretion, delays gastric emptying, and promotes satiety and reduced energy intake^28–30^. These multiple mechanisms of action underlie the remarkable efficacy of GLP-1 peptide analogues as therapies for diabetes and obesity^23,31^.

GLP-1 evolved to exert rapid transient effects to blunt postprandial glucose excursions, with a plasma half-life of a few minutes^32–34^, and is highly susceptible to enzymatic degradation^35–37^. Circulating GLP-1 is rapidly inactivated by the serum protease dipeptidyl peptidase-4 (DPP-IV), which removes the two *N*-terminal amino acids^26^, and neprilysin. GLP-1 also has numerous sites of proteolytic lability to the digestive proteases pepsin, trypsin, chymotrypsin and elastase (Fig. 1a)^38^. These enzymes digest protein-based foods to di- and tri-peptides for absorption in the small intestine, presenting a serious challenge to oral delivery^39^.

**Figure 1:**
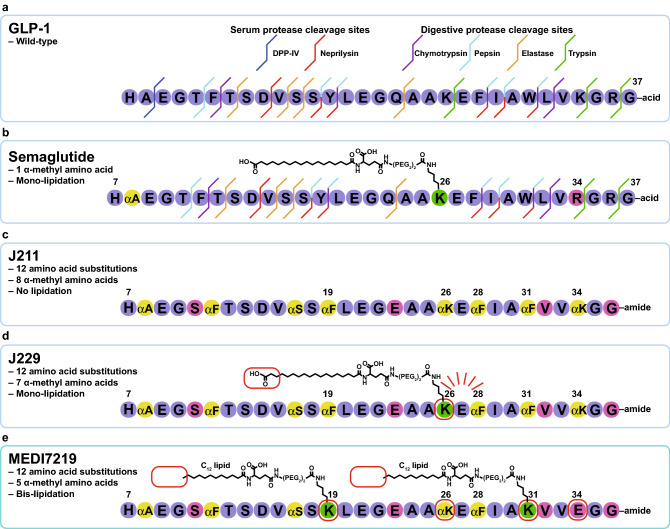
GLP-1RA design. (**a**) Amino acid schematic of wild-type GLP-1-(7–37) peptide showing protease cleavage sites. (**b**) Amino acid schematic of semaglutide showing amino acid substitution, site of lipidation and remaining protease cleavage sites. (**c**) Amino acid schematic of J211 showing amino acid substitutions. (**d**) Amino acid schematic of mono-lipidated J229. Red lines around positions 26–28 represent protection from trypsin provided by lipidation of Lys^26^. (**e**) Amino acid schematic of MEDI7219 peptide bis-lipidated with dodecanoic acid. Red boxes highlight differences compared with previous analogue for ***c–e***. *Amino acid numbering starts at 7 because active form of GLP-1 is cleaved from proglucagon to form GLP-1-(7 -37).

Previous approaches to engineering peptide therapeutics include substitution with unnatural amino acids to protect proteolytically vulnerable sites, and lipidation to promote reversible serum protein binding and reduced renal clearance^40–42^. Both of these approaches were considered in developing semaglutide to improve the circulating half-life. Ala^8^ is replaced with artificial α-methyl-L-alanine to protect the peptide against serum DPP-IV and the peptide is mono-lipidated at Lys^26,43^. Despite these modifications, semaglutide is still susceptible to rapid proteolytic degradation in the gastrointestinal tract^38^ (Fig. 1b). In the oral formulation, the permeation enhancer sodium N-(8-[2-hydroxybenzoyl]amino)caprylate (SNAC) is used to promote semaglutide absorption in the stomach and to provide some degree of protection against proteases in the stomach^44,45^. Although this can provide clinically meaningful systemic exposure, bioavailability is generally limited in the range of 0.4–1%^38,45^.

Here, we describe a systematic multidisciplinary approach to engineering novel oral peptide therapeutics based on the understanding of structure–activity relationships and controlled drug delivery, as applied to a series of peptidic GLP-1RAs designed for oral tablet administration. We aimed to achieve high oral bioavailability through high gastrointestinal stability, targeted release at the optimal absorption site, extended serum half-life appropriate for once-daily dosing, and full GLP-1RA potency. Staged progression from in vitro to increasingly relevant in vivo models enabled rational selection of a clinical lead based on these criteria. The anti-hyperglycaemic and weight- lowering action of this clinical candidate, designated MEDI7219, formulated as once-daily oral tablets, was evaluated and confirmed in a dog model of obesity and insulin resistance.

## Results

### Systemic and gastrointestinal stabilization of GLP-1 receptor agonists

#### Protection against proteolysis in serum and the gastrointestinal tract

To protect GLP-1 against both serum and gastrointestinal proteases, we modified the native peptide sequence by substituting in natural and α-methyl amino acids at predicted vulnerable sites (Fig. 1a). The aromatic residues Phe^12^, Tyr^19^, Phe^28^ and Trp^31^ are each targeted by chymotrypsin, pepsin and/or neprilysin, so were each replaced with α-methyl-L-phenylalanine (αMePhe). Introduction of αMePhe^12^ imposed steric restraints that hindered synthesis, which we reduced by replacing Thr^11^ with serine. A site of elastase vulnerability was removed by replacing Ser^17^ with α-methyl-L-serine (αMeSer). The basic residues Lys^26^ and Lys^34^ in native GLP-1 are targeted by trypsin, so were replaced with α-methyl-L-lysine (αMeLys). Furthermore, Arg^36^ was replaced with glycine to protect against tryptic cleavage. Protection against DPP-IV degradation was achieved by replacing Ala^8^ with α-methyl-L-alanine (αMeAla), as previously described for semaglutide^43^. To enhance aqueous solubility, Gln^23^ was replaced with glutamic acid, which also removed the potential for deamidation. Finally, replacement of Leu^32^ with valine provided a small but useful reduction in hydrophobicity that decreased proteolytic susceptibility of the Leu^32^-Val^33^ motif. This process yielded the peptide analogue J211 (Fig. 1c).

**Figure 2:**
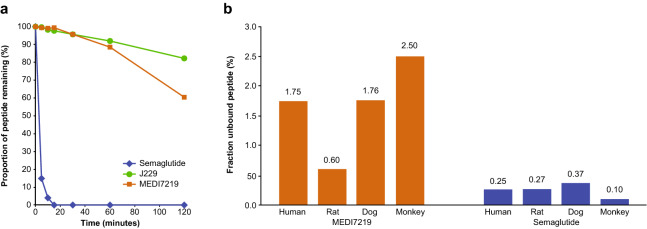
In vitro evaluation of GLP-1RAs. (**a**) FaSSIF incubation assay showing proportion of J229, MEDI7219 or semaglutide remaining after 2 h incubation with FaSSIF/pancreatin. (**b**) EScalate Equilibrium Shift Assay of protein binding showing binding propensity of MEDI7219 and semaglutide to human, dog, monkey and rat plasma protein.

To lipidate J211 for improved circulating half-life, we initially adopted the same approach as used for semaglutide^43^. Functionalizing J211 with Lys(Ɛ-(AEEA)_2_-γE-stearate) (C_18_ lipid dicarboxylate) at position 26 yielded mono-lipidated J229 (Fig. 1d). The proteolytic stability of J229 was dramatically improved compared with semaglutide: over 80% of J229 remained intact after 2 h of incubation in fasted-state simulated intestinal fluid (FaSSIF)/pancreatin, whereas semaglutide was completely degraded in less than 20 min (Fig. 2a). However, the in vitro potency of J229 was compromised compared with semaglutide and native GLP-1, with half maximal effective concentration (EC_50_) values of 132 pM versus 12 pM and 2.1 pM, respectively, in a cyclic adenosine monophosphate (cAMP) accumulation assay in Chinese hamster ovary (CHO) cells stably expressing human GLP-1R (Supplementary Fig. S1 and Table 1).

**Table 1:** cAMP accumulation assay in CHO cells stably expressing human GLP-1R (with 0.1% BSA or 4.4% HSA) or in EndoC-βH1 human pancreatic β-cells (with 0.1% BSA).

Peptide	CHO-hGLP-1R cAMP 0.1% BSA		CHO-hGLP-1R cAMP 4.4% HSA		Human EndoC-βH1 cAMP 0.1% BSA	
	EC_50_ (pM)	Effect at max. concentration (%)	EC_50_ (pM)	Effect at max. concentration (%)	EC_50_ (pM)	Effect at max. concentration (%)
GLP-1	2.1 (0.1)	102.8 (0.3)	3.3 (0.4)	105 (1.8)	120 (23)	100 (2.7)
Semaglutide	12 (2)	100.7 (0.9)	2,630 (461)	94.3 (1.8)	9,748 (3,283)	128.3 (5.8)
J229	132 (29)	100.0 (0.6)	Not tested	Not tested	58,310 (2709)	102.1 (7.7)
MEDI7219	3.4 (0.5)	101.7 (0.5)	398 (39)	94.3 (1.8)	1,365 (804)	97.5 (7.1)

Values are presented as geometric mean (standard error of mean). EC_50_ calculated from n ≥ 3 independent experiments.

BSA, bovine serum albumin; cAMP, cyclic adenosine monophosphate; CHO-hGLP-1R, Chinese hamster ovary cell line expressing human glucagon-like peptide 1 receptor; EC_50_, half maximal effective concentration; GLP-1, glucagon-like peptide 1; GLP-1R, glucagon-like peptide 1 receptor; HSA, human serum albumin.

#### Bis-lipidation of a GLP-1RA

We next scanned J211 for alternative lipidation sites to improve potency, and identified regions around αMePhe^19^ and αMePhe^31^. These residues were replaced with side-chain functionalized lysine residues, and the previously lipidated Lys^26^ was replaced with αMeLys. To reduce the number of α-methyl amino acids, αMeLys^34^ was replaced with glutamic acid. Functionalization of substituted Lys^19^ and Lys^31^ residues with dodecanoic acid yielded MEDI7219 (Fig. 1e). The proteolytic stability of MEDI7219 in FaSSIF/pancreatin was dramatically improved compared with semaglutide; with over 60% remaining intact after 2 h (Fig. 2a).

The in vitro potency of MEDI7219 was similar to semaglutide in the CHO cell cAMP accumulation assay in the presence of 0.1% bovine serum albumin (BSA), with low picomolar EC_50_ values for both peptides (MEDI7219, 3.4 pM; semaglutide, 12 pM; Supplementary Fig. S1). As anticipated, the potency of these peptides was reduced when a physiological level of 4.4% human serum albumin (HSA) was used in the CHO cell assay (MEDI7219, 398 pM; semaglutide, 2630 pM; Supplementary Fig. S1), but the EC_50_ difference between MEDI7219 and semaglutide was similar across assays. Potencies were comparable for both peptides when the assay was performed in the EndoC-βH1 human pancreatic β-cell line, which endogenously expresses GLP-1R (Supplementary Fig. S1). To confirm that bis-lipidation conferred a level of plasma protein binding expected to improve circulating half-life, we compared the peptides in an in vitro EScalate equilibrium shift assay. MEDI7219 and semaglutide both bound to human, dog, monkey and rat plasma proteins, with over 97% peptide bound in all cases. Higher unbound peptide proportions were observed for MEDI7219 than for semaglutide (Fig. 2b). Taken together, amino acid modification and bis-lipidation of MEDI7219 resulted in a proteolytically stable and potent peptide with bioactivity toward the GLP-1R in the presence of physiological HSA levels, supporting further development.

#### Potency of subcutaneously administered bis-lipidated peptides

**Figure 3:**
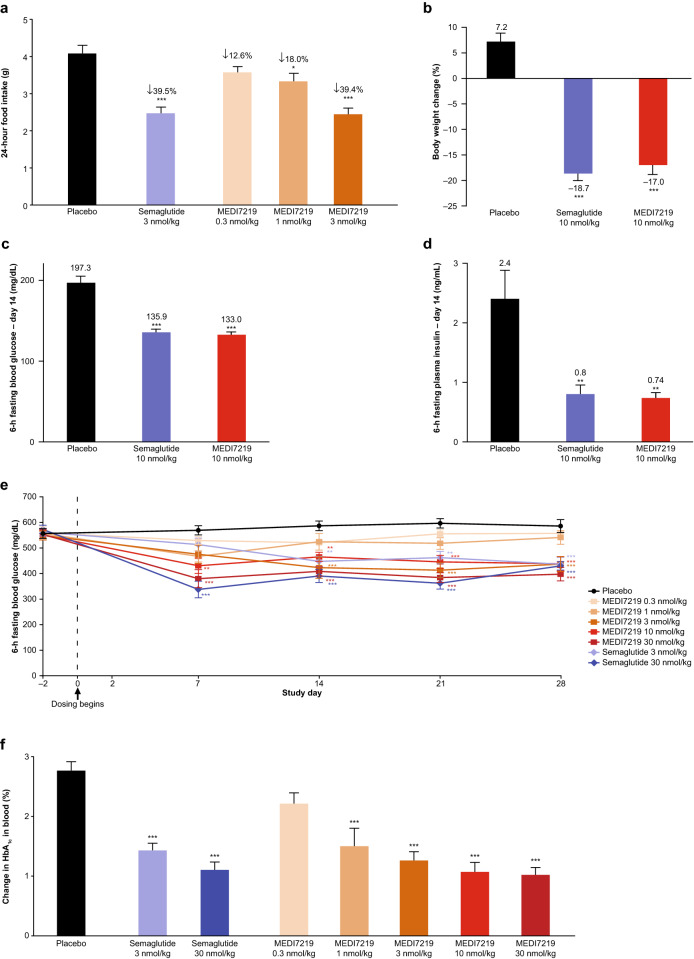
In vivo evaluation of GLP-1RAs. (**a**) Effect of subcutaneously administered MEDI7219 compared with semaglutide and placebo on food intake in the C57Bl/6 J mouse model (n = 6–9 per group). (**b**) Body weight change, (**c**) 6 h fasting glucose (day 14) and (**d**) 6 h fasting insulin levels (day 14) in DIO mouse model after repeated subcutaneous administration of MEDI7219, semaglutide or placebo over 21 days (n = 12 per group). (**e**) Blood glucose levels over 28 days in dose–response study in *db/db* mice treated with MEDI7219, semaglutide or placebo (n = 9 per group). (**f**) Change in %HbA_1C_ levels in 28-day dose–response study in *db/db* mice treated with MEDI7219, semaglutide or placebo (n = 9 per group). *P < 0.05, **P < 0.01, ***P < 0.001 versus placebo in all panels. Values are mean ± standard error of mean.

Having confirmed the in vitro properties of MEDI7219, we assessed in vivo potency in mouse models following subcutaneous administration. Single doses of MEDI7219 dose-dependently reduced food intake over 24 h in lean C57Bl/6 J mice, with the high dose (3 nmol/kg) having comparable effects (–39.4%, *P* < 0.001) to an equivalent dose of semaglutide (Fig. 3a). Lower doses of MEDI7219 also reduced food intake (1 nmol/kg, –18.0%, *P* < 0.05; 0.3 nmol/kg, –12.6%).

In the diet-induced obese (DIO) mouse model, MEDI7219 10 nmol/kg reduced body weight by 17.0% after 21 days, compared with an 18.7% reduction for semaglutide 10 nmol/kg and a 7.2% increase for placebo (Fig. 3b). Fasting glucose and insulin levels at day 14 were also reduced in DIO mice receiving once daily subcutaneous administration of MEDI7219 10 nmol/kg compared with placebo, and were similar to levels in mice receiving semaglutide 10 nmol/kg (Fig. 3c,d). In the diabetic *db*/*db* mouse model, MEDI7219 dose-dependently reduced glucose levels following once-daily subcutaneous administration for 28 days, with statistically significantly lower glucose levels at doses of 3–30 nmol/kg (*P* < 0.001) than placebo (Fig. 3e). Significant reductions in glucose levels were observed as early as day 7 in mice receiving MEDI7219 10 nmol/kg or 30 nmol/kg. MEDI7219 also dose-dependently reduced HbA_1C_ levels compared with placebo (*P* < 0.001 for doses 1–30 nmol/kg), with similar effects to those of semaglutide injected at doses of 3 nmol/kg and 30 nmol/kg (Fig. 3f).

### Selection of permeation enhancers for oral administration

**Figure 4:**
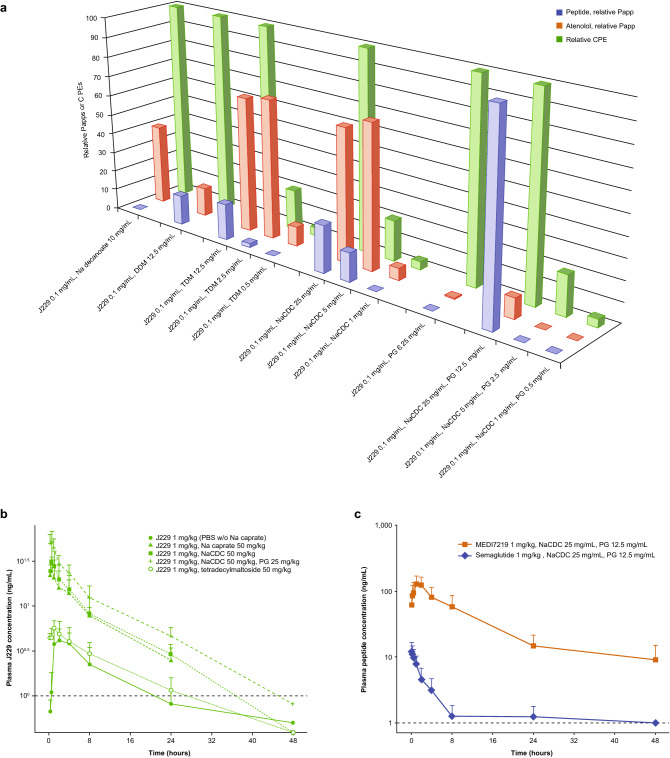
Oral tablet formulation development of GLP-1RAs. (**a**) Caco-2 screen for selection of permeation enhancers showing relative permeability coefficient of J229 (blue bars) compared with reference compound atenolol (orange bars) across Caco-2 monolayer in the presence of different permeation enhancers. Relative permeability expressed as percentage of highest observed apparent permeability coefficient (Papp; 43.1 × 10^–6^ cm s^-1^) of J229 0.1 mg/mL with NaCDC 25 mg/mL and PG 12.5 mg/mL. Green bars show relative concentration of permeation enhancers (CPE). (**b**) Mean plasma concentration–time profiles of J229 1 mg/kg in rats following single intraduodenal administration with permeation enhancers. (**c**) Mean plasma concentration–time profiles of MEDI7219 and semaglutide in rats following single intraduodenal administration of MEDI7219 with sodium chenodeoxycholate (NaCDC) and propyl gallate (PG) as permeation enhancers. Values are presented as mean and error bars represent standard error of mean (n = 4 per group). Lower limit of quantification (LLOQ; dotted line) is 1 ng/mL.

To optimize gastrointestinal absorption of our GLP-1 peptide analogues, we screened comprehensive panels of permeation enhancers (Supplementary Table S1) in vitro using Caco-2 cell monolayers (Fig. 4a) and in vivo using intraduodenal administration in rats (Fig. 4b). These experiments used mono-lipidated J229 as a model peptide with physicochemical characteristics similar to those of the bis-lipidated lead peptide MEDI7219. The in vitro screen identified a novel combination of sodium chenodeoxycholate (NaCDC) and propyl gallate (PG) as the optimal permeation enhancers in the panel tested (Fig. 4a). In rats, the largest improvement in bioavailability following intraduodenal administration was with 50 mg/kg NaCDC and 25 mg/kg PG, among the panel tested. This combination increased bioavailability to a systemic fraction (%F) of 0.39 compared with 0.02 for J229 alone, at a dose of 1 mg/kg (Fig. 4b). Switching to MEDI7219, mean bioavailability was 13-fold higher than for semaglutide when administered intraduodenally with permeation enhancers in rats (%F, 1.01 vs 0.08) (Fig. 4c).

### Site of gastrointestinal absorption of stabilized GLP-1RA peptides in dogs

We used IntelliCap controlled release capsules^46^ to determine the site of maximal gastrointestinal absorption of our stabilized GLP-1RAs in dogs. Capsules filled with J229 formulated with permeation enhancers were actuated at various pH-dependent points along the gastrointestinal tract following oral administration (Table 2). Bioavailability of J229 was highest when released in the proximal colon (%F, 3.8) and the small intestine (%F, 2.2), but was low following oral gavage of the same liquid formulation used in the capsules (%F, 0.2). IntelliCap capsule transit times through each compartment of the gastrointestinal tract were recorded, and the effect of site of peptide release was investigated. When J229 was released in the small intestine, transit times through site of the release and through downstream compartments were slowed (Supplementary Table S2). Therefore, we aimed to formulate MEDI7219 tablets for peptide delivery to sites of maximal absorption in the small intestine and proximal colon.

**Table 2:** Pharmacokinetic parameters of J229 delivered via different routes in dogs to determine site of absorption.

	Group				
	1	2	3	4	5
J229 dose, mg/kg	0.74	0.74	0.74	0.01	0.01
Formulation	J229 8 mg/mL, NaCDC 10 mg/mL, Na caprate 2 mg/mL	J229 8 mg/mL, NaCDC 10 mg/mL, Na caprate 2 mg/mL	J229 8 mg/mL, NaCDC 10 mg/mL, Na caprate 2 mg/mL	J229 0.1 mg/mL	J229 0.1 mg/mL
Dosing route	Oral gavage	Oral IntelliCap capsule – proximal small intestine	Oral IntelliCap capsule – proximal colon	Intravenous	Subcutaneous
T_max_, median (range) – hours	4 (2–8)	2 (0.5–24)	0.25 (0.25–1.0)	0.25 (0.08–0.5)	24 (8–48)
C_max_, ng/mL	8.2 (32.6)	228.0 (59.3)	237.0 (85.3)	162.0 (15.2) (27.8)	52.7 (14.7)
AUC_last_, ng·h/mL	249.0 (27.2)	2,270.0 (40.0)	3,970.0 (81.1)	1,410.0	1,880.0 (19.4)
F, %	0.2 (27.2)	2.2 (40.0)	3.8 (81.1)	100.0 (27.8)	133.3 (19.4)

Values are presented as arithmetic mean (% coefficient of variation) unless otherwise stated, n = 5 per group.

AUC_last_, area under the curve from time 0 to last measurable concentration; C_max_, maximum serum concentration; F, bioavailability (systemic fraction); NaCDC, sodium chenodeoxycholate; T_max_, time to reach C_max_.

### Oral bioavailability of MEDI7219 tablets

We formulated MEDI7219 as enteric-coated oral tablets containing 20 mg of peptide with 300 mg of permeation enhancers (100 mg of NaCDC and 200 mg of PG) for pharmacokinetic studies in dogs. The enteric coating was chosen to protect the tablet from the low pH of the stomach, and to release the drug by dissolution in the neutral pH of the intestine. For comparison, semaglutide was formulated as uncoated tablets containing 20 mg of peptide and 300 mg of SNAC permeation enhancer. After oral administration, the mean bioavailability of MEDI7219 was considerably higher than that of semaglutide (%F, 5.92 vs 0.08) and the mean plasma half-life of MEDI7219 was shorter than that of semaglutide (9.8 h vs 60.5 h) (Table 3). These half-life values were consistent with the lower levels of in vitro plasma protein binding already observed for MEDI7219 compared with semaglutide (Fig. 2b). These findings confirmed pharmacokinetic parameters suitable for once-daily oral dosing of MEDI7219 in this tablet formulation.

**Table 3:** Pharmacokinetic parameters of MEDI7219 and semaglutide oral tablets in dogs.

	Peptide	
	Semaglutide	MEDI7219
Peptide dose, mg/dog	20	20
PE dose, mg/dog	300 (SNAC)	300 (100 mg NaCDC and 200 mg PG)
Coating	none	pH 5.5 enteric
t_½_, hours	60.5 (13.6)	9.8 (13.2)
T_max_, hours	2.0 (1.5–2.5)	1.5 (1.0–4.5)
C_max_, ng/mL	21.1 (72.5)	1,450 (63.2)
AUC_0-inf_, ng·h/mL	1,440 (45.8)	13,500 (54.2)
F, %	0.08 (45.8)	5.92 (54.2)
CL/F, mL/h	16,000 (47.4)	1,880 (52.1)

Values are presented as arithmetic mean (% coefficient of variation), except T_max_ shown as median and range (min–max), n = 9 per group.

AUC_0-inf_, area under the curve from time 0 to infinity; CL/F, oral clearance; C_max_, maximum serum concentration; F, bioavailability (systemic fraction); NaCDC, sodium chenodeoxycholate; PE, permeation enhancer; PG, propyl gallate; SNAC, sodium N-(8-[2-hydroxybenzoyl]amino)caprylate; t_½_, half-life; T_max_, time to reach C_max_.

### Effects of MEDI7219 oral tablets on weight and glucose control in a dog model of obesity and insulin resistance

**Figure 5:**
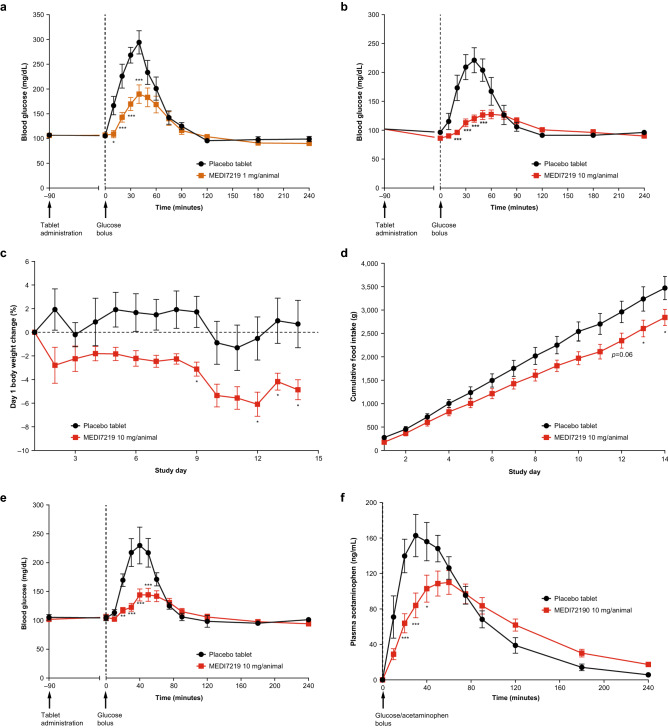
Pharmacodynamic effects of MEDI7219 in HFHF fed dogs. Oral glucose tolerance test in HFHF fed dogs after treatment with a single (**a**) 1 mg/animal or (**b**) 10 mg/animal oral dose of MEDI7219 or placebo tablets (n = 7–11 per group). (**c**) Change in body weight over 14 days in HFHF fed dogs treated with a daily oral dose of MEDI7219 10 mg/animal or placebo tablets. (**d**) Cumulative food intake over 14 days of HFHF fed dogs treated with a daily oral dose of MEDI7219 10 mg/animal or placebo tablets. (**e**) Day 14 oral glucose tolerance test in HFHF fed dogs treated with a daily oral dose of MEDI7219 10 mg/animal or placebo tablets. (**f**) Effect of MEDI7219 on gastric emptying at day 14 in HFHF fed dogs treated with a daily oral dose of MEDI7219 10 mg/animal or placebo, as shown by plasma acetaminophen levels monitored after administration of acetaminophen with glucose bolus during glucose tolerance testing. *P < 0.05, **P < 0.01, ***P < 0.001 versus placebo in all panels. Values are mean ± standard error of mean (MEDI7219, n = 12 per group; placebo, n = 6 per group in the multiple-dose study).

The potential anti-diabetic efficacy of MEDI7219 oral tablets was evaluated in dogs with obesity, insulin resistance and mild hyperglycemia induced by a high-fructose/high-fat (HFHF) diet^47,48^. Plasma glucose excursions were statistically significantly improved 10–40 min after an oral glucose challenge following single doses of MEDI7219 1 mg or 10 mg oral tablets, compared with placebo (*P* < 0.05; Fig. 5a,b). Over 14 days, dogs receiving once-daily MEDI7219 10 mg oral tablets showed significantly higher body weight loss than those receiving placebo (–4.86% vs + 0.71%, *P* < 0.05; Fig. 5c). This correlated with significantly decreased cumulative food intake (*P* < 0.05) over the 14-day period (Fig. 5d). An oral glucose tolerance test performed in fasted dogs postdose on day 14 also revealed significant improvements in glucose excursions in MEDI7219-treated dogs compared with placebo control (*P* < 0.01; Fig. 5e).

To investigate the potential effect of oral MEDI7219 on gastric emptying, a typical GLP-1R mediated effect^49^, an oral dose of acetaminophen, which is absorbed in the small intestine^50^, was administered during the glucose tolerance test in dogs on day 14. Peak plasma acetaminophen concentration was significantly reduced at 20–40 min (*P* < 0.05) and delayed in the MEDI7219 group compared with the placebo group (Fig. 5f), indicating that MEDI7219 treatment led to a delay in gastric emptying.

## Discussion

Oral delivery of peptide therapeutics represents a significant advance in the ability to administer medicines in a convenient and patient-friendly way, with the potential to improve adherence and consequently treatment outcomes. Overcoming the challenges of proteolytic degradation, gastrointestinal permeability, and rapid renal clearance to ensure high bioavailability, whilst maintaining biological function, requires a rational design process. We have presented a template for this process using a systematic multidisciplinary approach to develop a novel GLP-1RA peptide therapeutic that exhibited potent agonism, a circulating half-life suitable for once-daily dosing and significant oral bioavailability. The improved absolute oral bioavailability of MEDI7219, ~ 6% in dogs, was achieved without compromising potency by stepwise engineering based on structure–activity relationships.

The rational approach in the design and development of MEDI7219, starting with engineering the peptide to remove sites of vulnerability to both serum and gastrointestinal proteases through amino acid substitution, contrasts with previous approaches to oral peptide delivery, which have relied on retrofitting injectables to the oral route without stabilizing peptide sequences against gastrointestinal proteases. Furthermore, enteric-coated MEDI7219 tablets were designed to withstand transit through the stomach releasing the peptide at the site of maximal absorption in the small intestine, which was identified with the use of the IntelliCap system. Taken together, the extensive screening identified an effective combination of permeation enhancers that greatly improved the inherently low uptake of a peptide from the gastrointestinal tract, resulting in high oral bioavailability (~ 6%) of MEDI7219 in dogs.

MEDI7219 is the first bis-lipidated GLP-1RA peptide, and this bis-lipidation approach was crucial for promoting plasma protein binding to reduce renal clearance without sacrificing agonist activity, while minimizing the use of α-methyl amino acids to stabilize the molecule. The pair of C_12_ lipids in MEDI7219 conferred a shorter circulating half-life than the single C_18_ lipid dicarboxylic acid in semaglutide when tested in dogs. This correlated to plasma protein binding data, in which a higher fraction of unbound MEDI7219 than semaglutide was observed for plasma proteins of all species tested. Although the half-life of MEDI7219 is shorter than that of semaglutide in dogs, allometric scaling (Supplementary Fig. S2) supports once-daily dosing in humans (t_½_ 15 h).

In a dog model of obesity and insulin resistance, once-daily administration of MEDI7219 oral tablets led to both decreased glucose excursion, when challenged with an oral glucose bolus, and body weight loss, consistent with the action of other GLP-1RA peptide therapeutics^23,27^. Administration of acetaminophen during the oral glucose challenge indicated that MEDI7219 delayed gastric emptying, as expected with GLP-1R agonism^51–53^, and this also probably contributed to the decreased glucose levels. These findings confirmed that we achieved a preclinical efficacy profile for MEDI7219 supportive of progression to clinical trials.

The average bioavailability of MEDI7219 was approximately fivefold higher than the published value for semaglutide in dogs (%F, 5.92 vs 1.22)^45^. We observed lower semaglutide bioavailability with our in-house tablets than published values, resulting in an even greater magnitude of improvement in bioavailability for MEDI7219. This suggests that some methodological differences in the manufacture of oral semaglutide tablets may have led to lower bioavailability than that reported in previously published data. Nevertheless, our results confirm a dramatic and unprecedented high oral bioavailability for MEDI7219 compared with other linear peptide therapeutics.

The dog model used to test the activity of MEDI7219 had advantages for the in vivo characterization because it allowed for oral administration of tablets, which is not possible in rodents. In addition, because dogs gained excess body weight on a high-fat diet and became insulin resistant, it was possible to ascertain GLP-1R-mediated pharmacological effects in the model following repeated oral dosing. Pharmacokinetic parameters are influenced by characteristics such as gastrointestinal barriers and plasma protein binding, and may vary among species. Furthermore, interspecies physiological differences, such as gastrointestinal pH values and transit time, may also affect formulation performance. Bioavailability variability was ~ 50% coefficient of variation in the well-controlled dog study but may be different in a clinical population, potentially affecting the therapeutic window. Therefore, it is crucial to consider potential translational difference when moving from dog to human studies, and to monitor these concerns closely in the clinical setting.

In conclusion, here we demonstrate the very first peptide synthesized by biotechnology routes to address the intrinsic limitation of oral peptide delivery and first pass effects. Our data demonstrated reasonable bioavailability in preclinical models and therefore a very robust approach when coupled with the site of absorption and controlled drug delivery. Our systematic multidisciplinary approach for engineering a novel oral GLP-1RA offers a valuable model for the development of future oral peptide therapeutics for diabetes and other chronic conditions. This has the potential to make these peptides more accessible to patients worldwide, with the opportunity for improved patient adherence and hence reduced hospitalization or other side effects resulting from non-compliance. We leveraged advances in peptide chemistry and solid dosage formulation to develop a once-daily oral tablet GLP-1RA with high bioavailability, and demonstrated efficacy in a preclinical model of insulin resistance and obesity. Our findings warrant clinical studies of MEDI7219 to confirm translatability of observed bioavailability and pharmacology from dogs to humans.

## Materials and methods

### In vivo studies

The development program involved peptide optimization using in vitro models, and in vivo potency testing in mouse models and formulation testing using rat models. Methods are reported in accordance with the Animal Research: Reporting of In Vivo Experiments (ARRIVE) guidelines. Animal studies were conducted at AstraZeneca (Gaithersburg, MD, USA or Cambridge, UK), Charles River Laboratories (Shrewsbury, Wilmington or Worcester, MA; Mattawan, MI, USA) or Covance Laboratories (Madison, WI, USA). The study was conducted in accordance with the Animals (Scientific Procedures) Act 1986, under a Project Licence reviewed by the establishment Animal Welfare and Ethical Review Body (AWERB) and granted by the UK Home Office. Study protocols were approved by the Institutional Animal Care and Use Committee at AstraZeneca (Gaithersburg, MD, USA), Charles River Laboratories (Shrewsbury, Wilmington or Worcester, MA; Mattawan, MI) or Covance Laboratories (Madison, WI, USA), and were in compliance with national laws and regulations ensuring humane use and care of laboratory animals and the AstraZeneca Animal Welfare and Bioethics policies. In vivo studies were not blinded, confounders were not controlled for, and no criteria were set for including or excluding animals or data points from analyses.

### Systemic and gastrointestinal stabilization of GLP-1 receptor agonists

#### Amino acid substitution and lipidation

*N*-α-Fmoc-L-amino acids were from Bachem (Bubendorf, Switzerland), and α-methyl and other unnatural amino acids were from Iris Biotech (Marktredwitz, Germany), Pharmaron (Beijing, China) or PepTech (Burlington, MA, USA); solvents were from Merck (Darmstadt, Germany). Peptides J211, J229 and MEDI7219 (Fig. 1) were prepared as C-terminal carboxamides on Novabiochem NovaSyn TentaGel Rink (Merck) synthesis resin using standard chemistry and coupling procedures^54^, and reagents from Sigma-Aldrich (Gillingham, UK). Amino acids following α-methyl residues were coupled twice to ensure full incorporation. The *N*-terminal histidine residue of GLP-1 was incorporated as Boc-His(Trt)-OH to simplify peptide cleavage. At designated lipidation positions, Fmoc-L-Lys(Mmt)-OH was incorporated into the peptide backbone during automated assembly and the Mmt protecting groups were removed upon completion. The acidified resin was quenched, and the exposed epsilon amino functions were selectively lipidated as required before peptide cleavage. Crude peptides were purified chromatographically using 5 µm Agilent Polaris C8-A (mono-lipidated; Agilent, Santa Clara, CA, USA) or Waters XBridge C18 stationary phases (bis-lipidated; Waters, Antwerp, Belgium), and lyophilized. Purified peptides were characterized by single quadrupole liquid chromatography/mass spectrometry (LC/MS) with a Waters XBridge C18 stationary phase, using a generic linear binary gradient of 10–90% methyl cyanide (MeCN; 0.1% trifluoroacetic acid [TFA] v/v) in water and the Waters MassLynx 3100 platform (ESI^+^ mode, monitoring 3 M + H and 4 M + H ions) to verify molecular mass. Analytical reverse-phase high-performance liquid chromatography (RP-HPLC) was conducted using an Agilent Polaris C8-A stationary phase (3 µm) at 1.5 mL min^-1^ with a linear binary gradient of 10–90% MeCN (0.1% TFA v/v) in water, and monitored by UV absorption at 210 nm. Overall yields of J211, J229 and MEDI7219 were greater than 50% based on initial resin functionalisation (0.24 mmol/g). Mass spectrometry data for all three peptides was consistent with calculated values (given below).

J211

- Requires: 3228.60 Da: 2 M^+2^ = 1615.30, 3 M^+3^ = 1077.20, 4 M^+4^ = 808.15
- Found: 2 M^+2^ = 1615.55, 3 M^+3^ = 1077.15, 4 M^+4^ = 808.10

J229

- Requires: 3930.45 Da: 2 M^+2^ = 1966.23, 3 M^+3^ = 1311.15, 4 M^+4^ = 983.61
- Found: 2 M^+2^ = 1966.34, 3 M^+3^ = 1311.25, 4 M^+4^ = 983.75

MEDI7219

- Requires: 4352.99 Da: 3 M^+3^ = 1451.99, 4 M^+4^ = 1089.25
- Found: 3 M^+3^ = 1451.89, 4 M^+4^ = 1089.28

See Supplementary data for additional LC/MS information.

#### Fasted-state simulated intestinal fluid/pancreatin assay

Fresh FaSSIF and USP Pancreatin (FaSSIF/p; Sigma-Aldrich) was prepared according to Galia *et al*^55^. and used immediately. Peptides were dissolved in pre-warmed FaSSIF before addition of pre-warmed FaSSIF/pancreatin with agitation. Samples were analyzed by analytical RP-HPLC to determine remaining intact peptide by area under the curve.

#### EScalate equilibrium shift assay

Binding of peptides to human plasma proteins was determined by the EScalate equilibrium shift assay (Sovicell, Leipzig, Germany), using five dilutions of plasma in phosphate buffered saline and an assumed binding protein concentration of 600 µM. Samples were incubated for 1 h at room temperature then centrifuged to remove the HSA-coated beads. Supernatant samples were analyzed using HPLC (3 µm Phenomenex Aeris Widepore XB-C18 stationary phase) coupled to an electrospray ionization quadrupole time-of-flight mass spectrometer in high resolution accurate mass mode (Agilent, Santa Clara, CA, USA). Unbound fractions were calculated from the concentration-dependent shift in binding equilibrium according to ${f}_{u}=\frac{1}{1+\frac{P}{{K}_{D}^{Plasma}}}$, where $P$ is the concentration of the binding protein in plasma and ${K}_{D}^{Plasma}$ is the dissociation constant of the compound from plasma proteins in solution.

#### Cyclic adenosine monophosphate accumulation assay

All reagents for cAMP assays were obtained from Sigma-Aldrich unless otherwise specified. CHO-K1 cells (ATCC) were stably transfected with a human GLP-1R expression plasmid and maintained in Dulbecco’s Modified Eagle Medium, 10% fetal bovine serum, 500 μg/mL geneticin and 400 μg/mL hygromycin B. Human EndoC-βH1 cells were kindly provided by Professor Raphael Scharfmann (Endocells, Paris, France), and were maintained in Dulbecco’s Modified Eagle Medium (low glucose), 2% BSA fraction V (Roche Diagnostics, Basel, Switzerland), 50 µM 2-mercaptoethanol, 10 mM nicotinamide, 5.5 µg/mL transferrin and 6.7 ng/mL sodium selenite.

Peptide serial dilutions were prepared in assay buffer (Hank’s Balanced Salt Solution [HBSS] containing 25 mM HEPES and 0.5 mM IBMX; pH 7.4) supplemented with 0.1% BSA or 4.4% HSA, using an Echo 550 acoustic liquid handler (Labcyte Inc., Sunnyvale, CA, USA) to obtain an 11-point concentration–response curve. Cells were suspended in assay buffer and combined with serially diluted peptides at room temperature for 30 min. cAMP levels were measured using a cAMP dynamic 2 HTRF kit (Cisbio, Codolet, France), following the manufacturer’s two-step protocol, on an EnVision plate reader (PerkinElmer, Waltham, MA, USA). Data were transformed to % Delta F, as described in the manufacturer’s guidelines, and expressed as % activation, in which 80 nM native GLP-1 peptide (Bachem) defines maximum effect. The transformed data were analyzed by four-parameter logistic fit to determine EC_50_ values using GraphPad Prism 6 (GraphPad Software, San Diego, CA, USA).

### In vivo potency of subcutaneously administered bis-lipidated peptides

#### Acute food intake in C57Bl/6J mice

Male C57Bl/6 J mice 8–10 weeks of age (Jackson Laboratories, Bar Harbor, ME, USA) were single-housed in the BioDAQ (Research Diets, New Brunswick, NJ, USA) food monitoring system with ad libitum access to standard chow and water. Mice were randomized into groups of 6–9 per group on baseline 24-h food intake. On the first study day, mice were fasted for 6 h and then received a single subcutaneous dose of test peptide or placebo (50 mM Tris–HCl, 150 mM mannitol, 0.02% polysorbate 80; pH 8.0). Food intake was monitored over the next 48 h.

#### Body weight, blood glucose and plasma insulin in diet-induced obese mouse model

Male 20-week-old C57Bl/6 J mice were single-housed for approximately 14 weeks with ad libitum access to water and 60% high-fat diet (D12492, Research Diets). Mice were randomized into groups of 12 mice per group on baseline body weight, 6 h fasting glucose and 6 h fasting insulin. Over 21 days, mice received daily subcutaneous injections of 10 nmol/kg of test peptides or placebo (50 mM Tris–HCl, 150 mM mannitol, 0.02% polysorbate 80; pH 8.0). Body weight was measured daily and fasting glucose and insulin levels were measured on day 14 following a 6-h fast. Blood was collected via tail-snip and glucose was measured with an Ascensia Breeze 2 glucometer (Bayer, Mishawaka, IN, USA). Insulin was measured in plasma using a Meso Scale rat/insulin kit (Meso Scale Discovery, Rockville, MD, USA).

#### Dose–response study of MEDI7219 in diabetic db/db mouse model

Male 7-week-old *db*/*db* mice (Charles River, Bristol, UK) were group-housed with ad libitum access to standard chow and water. Mice were randomized to groups of nine mice per group on baseline body weight, HbA_1C_ and 4-h fasting glucose (assessed in tail bleed samples using a Cobas c-111 analyzer [Roche Diagnostics, Indianapolis, IN, USA] and Nova StatStrip Xpress glucometer [DSI, St. Paul, MN, USA]). Insulin was measured in plasma from tail bleeds using a Meso Scale kit. Over 28 days, mice received daily subcutaneous doses of test peptides or placebo (50 mM Tris–HCl, 150 mM mannitol, 0.02% polysorbate 80; pH 8.0).

### Selection of permeation enhancers for oral administration

#### Caco-2 screen

Caco-2 cells were trypsinized, suspended in medium and seeded to wells of a Millipore 96-well plate following standard procedures^56^. Cells were fed at 2-day intervals for 3 weeks until a transepithelial electric resistance of ~ 1000 ohms/cm^2^ was achieved. Test samples containing peptide and permeation enhancers (Supplementary Table S1) were prepared in HBSS solution (pH 7.4). Lucifer Yellow and atenolol were added to the test samples as internal controls. The cells were incubated for 3 h with test samples on the apical side and blank media on the basolateral side. Media from the apical and basolateral sides were collected after 3 h and peptide content was evaluated^57^. Data are expressed as apparent permeability (Papp):

$$Papp=\frac{dQ}{dt}\cdot \frac{1}{\left(A \cdot {C}_{0}\right)}$$

where $dQ/dt$ is the rate of permeation, ${C}_{0}$ is the initial concentration of test agent, and $A$ is the area of the monolayer.

#### *Oral bioavailability of GLP-1RAs with permeation enhancers *in vivo

Male Sprague Dawley rats (Charles River, Shrewsbury, MA, USA) weighing 250–275 g were housed with ad libitum access to standard chow and water. Test peptides were administered via either an intravenous or a duodenal catheter (4 rats per group) after overnight fasting, and food was returned following 4 h blood collection. Blood was collected via a jugular vein catheter at − 5, 15 and 30 min and 1, 2, 4, 8 and 24 h after intravenous dosing, or for intraduodenal groups at 15 min predose and 0.5, 1, 2, 4, 8, 24 and 48 h postdose. Blood was collected in K_2_EDTA tubes and kept on wet ice prior to 10 min centrifugation (3000 × *g*) at 5 °C ± 3 °C. Plasma samples were stored at − 70 °C until analysis.

### Site of gastrointestinal absorption of stabilized GLP-1RA peptides in dogs

Fasted male beagle dogs weighing 6–8 kg were pretreated with pentagastrin 6 μg/kg. After 30 min, dogs (5 per group) were administered with either: IntelliCap capsules (Medimetrics, Eindhoven, Netherlands) filled with 0.7 mL of peptide and permeation enhancer solution (J229 8 mg/mL, NaCDC 12 mg/mL); 0.7 mL equivalent oral gavage and 20 mL gavage rinse; J229 0.1 mg/mL intravenous injection; or 0.1 mg/mL subcutaneous injection. Blood was collected at 0.25, 0.5, 1, 2, 4, 8, 24 and 48 h for subcutaneous and oral gavage groups. Blood was collected at 0.083, 0.25, 0.5, 1, 2, 4, 8 and 24 h for the intravenous group. Blood collection in IntelliCap groups started after actuation of capsule content release. Blood was collected from the jugular vein in K_2_EDTA tubes and kept on wet ice. Plasma samples were stored at − 60 °C to − 90 °C until analysis.

### Oral bioavailability of MEDI7219 tablets

#### Oral tablet formulation

Tablets contained 0.5–10 mg of MEDI7219, 150 mg of permeation enhancers (50 mg NaCDC [Prodotti, Basaluzzo, Italy] and 100 mg PG [Guangzhou Hanfang Pharmaceutical, Guangzhou, China]), mannitol (Roquette, Keokuk, IA, USA) as filler, crospovidone (BASF, Ludwigshafen, Germany) as disintegrant, sodium stearyl fumarate (JRS Pharma, Rosenberg, Germany) as lubricant and hydrophobic fumed silica (Evonik, Parsippany, NJ, USA) as glidant, with a total weight of 245 mg per tablet. Placebo tablets were identical to active tablets, except for the absence of test peptide. Tablets were made by: sieving ingredients; blending in a Turbula mixer (WAB, Muttenz, Switzerland); dry granulation by compression in a pellet machine (Parr Instrument, Moline, IL, USA) and sieving (1 mm); blending with lubricant and glidant in a Turbula mixer; and compression at 50 N hardness on an NP-RD10 tablet press (Natoli, St Charles MO, USA). Finally, tablets were enteric coated with Eudragit polymer suspension (Evonik, Darmstadt, Germany) to achieve a 12.5% weight gain using a Vector LDCS3 pan coater (Freund-Vector, Marion, IA, USA).

#### Oral bioavailability of MEDI7219 in dogs

Fasted male beagle dogs weighing 8–13 kg (5 per group) were administered with enteric-coated oral tablets containing 10 mg test peptide and 300 mg permeation enhancers were administered. Blood samples were collected at 0.5, 0.5, 1, 1.5, 2, 2.5, 3.5, 4.5, 8, 24 and 48 h postdose.

### Effect of MEDI7219 oral tablets on weight and glucose control in a dog model of obesity and insulin resistance

Eighteen male beagle dogs weighing an average of 10.9 ± 0.3 kg were housed at Charles River Laboratories (Worcester, MA, USA) and received two cups of 5A4J HFHF canine diet daily (20.5% protein, 52.9% fat, 26.6% carbohydrate, 18.9% fructose; ScottPharma Solutions, Marlborough, MA, USA) plus water ad *libitum*. For the single-dose study, dogs were randomized on baseline glucose levels to receive MEDI7219 1 mg or 10 mg or placebo tablets in the morning following an overnight fast (7–11 dogs per group). Ninety minutes after tablet administration, dogs received an oral glucose bolus (200 mg/mL at a 5 mL/kg volume calculated on body weight prior to HFHF feeding), and blood glucose (blood collected via peripheral vein) was measured with an AlphaTRAK glucometer system (Zoetis, Parsippany, NJ, USA) at –90, 0, 10, 20, 30, 40, 50, 60, 75, 90, 120, 180 and 240 min after glucose administration. Approximately 12 weeks later, dogs entered the multiple-dose study and received either MEDI7219 10 mg (n = 12) or placebo tablets (n = 6) once daily for 14 days. Dogs received two cups of HFHF canine diet per day, with feeding 3 h postdose during the 14-day study. Dogs had access to water ad libitum throughout the study. Food intake and body weight were monitored daily. On day 14, after overnight fasting, dogs received their final dose followed by an oral glucose and acetaminophen bolus 90 min after administration. Blood glucose was measured as described previously, and plasma acetaminophen levels were measured by LC/MS method at the same time points.

### Quantification of plasma concentrations of peptides

Fifty or seventy μL aliquots of K_2_EDTA plasma samples were precipitated with 75% acetonitrile (Sigma-Aldrich) in water (J.T. Baker, Phillipsburg, NJ, USA) (v/v) and centrifuged at 2000 × *g*. The supernatant was dried under nitrogen at 60 °C, then cooled. Samples were reconstituted in 20% acetonitrile in water (v/v). The extracted samples were separated using a Waters Acquity UPLC BEH C18 column (2.1 × 100 mm) on a Shimadzu Nexera UHPLC at 60 °C with 0.7 mL/min flow rate, and detected using a SCIEX TripleTOF 6600 operating in full scan + MS2 positive ion mode or a SCIEX 5500 operating in MRM positive ion mode, or a Thermo TSQ Vantage operating in MRM mode. Gradient separation was performed with water and 0.2% formic acid (Thermo Fisher Scientific, Waltham, MA, USA) as mobile phase A, and acetonitrile with 0.2% formic acid as mobile phase B. The method was qualified for the quantification range of 1–1000 ng/mL with accuracy and precision of ± 20%, except for at the lower limit of quantification when the accuracy and precision were ± 25%. Plasma concentrations were subject to noncompartmental analysis consistent with the route of administration using Phoenix WinNonlin (version 7.0, Certara, Princeton, NJ, USA).

### Statistical analyses

Sample sizes were calculated based on power analyses for pharmacodynamic endpoints. Statistical analyses were performed using GraphPad software (San Diego, CA, USA). Results are expressed as mean ± standard error of the mean unless otherwise stated. In vivo data were analyzed with one-way analysis of variance (ANOVA) followed by Tukey post hoc analysis (acute food intake, *db*/*db* HbA_1C_ data). Longitudinal data were analyzed by two-way ANOVA followed by Tukey post hoc analysis (*db*/*db* glucose and DIO mouse data) or two-way ANOVA followed by Sidak or Bonferroni post hoc analysis (HFHF canine acute oral glucose tolerance test data and 14-day data). In all statistical tests *P* < 0.05 was considered significant. One animal in the single MEDI7219 10 mg dose group for the glucose tolerance test was excluded from analysis due to a likely error in dosing. In the analysis of the effect of MEDI7219 on body weight in HFHF fed dogs, one animal in the placebo group and one datapoint on day 10 in the MEDI7219 group were excluded due to errors in measurement. No other animals or data points were excluded from any of the in vivo study analyses.

## Supplementary Information

Supplementary Information.
