# Supplementary material for: Development of an orally delivered GLP-1 receptor agonist through peptide engineering and drug delivery to treat chronic disease
Source: Sci Rep. 2021 Nov 18;11:22521. doi: 10.1038/s41598-021-01750-0 (PMC8602401; doi:10.1038/s41598-021-01750-0)

# Supplementary data

## Supplementary Table S1. List of permeation enhancers combined with J229 for intraduodenal pharmacokinetics studies in rats.

| **Permeation enhancers** | |
| --- | --- |
| Sodium decanoate (Na caprate) | Octyl gallate |
| Sodium octanoate (Na caprylate) | Lauryl gallate |
| Dodecylmaltoside (DDM) | Sodium N-(8-[2-hydroxybenzoyl]amino)caprylate (SNAC) |
| Tetradecylmaltoside (TDM) | Palmitoyl carnitine |
| Sodium chenodeoxycholate (NaCDC) | Polyoxyl 15 hydroxystearate (Kolliphor HS 15) |
| Sodium deoxycholate | Polyoxyethylene (10) oleyl ether (Brij 10) |
| Sodium tetraglycocholate | **Polyethylene glycol hexadecyl ether** |
| Sodium ursodeoxycholate | **Polyoxyethylene (10) cetyl ether** |
| Sodium glycocholate | **Purebright** |
| Sodium taurocholate | **Diethylene glycol monoethyl ether (Transcutol ES)** |
| Sodium cholate | **Polyoxyl-35 castor oil (Kolliphor EL)** |
| Chenodeoxycholic acid | **Lauroglycol FCC** |
| Ethyl gallate | **Trimethyl chitosan** |
| Propyl gallate (PG) | Chitosan |

## Supplementary Table S2. Site of GLP-1RA absorption – transit time through gastrointestinal tract after J229 release from IntelliCap.

| Capsule actuation site | Transit time | | | |
| --- | --- | --- | --- | --- |
|  | **Stomach** hh:mm | **Small intestine** hh:mm | **Colon** hh:mm | **Whole gut** hh:mm |
| Proximal small intestine | 01:30 (00:21–03:22) | 07:28 (05:39–10:09) | 09:59 (01:59–17:19) | 18:58 (08:53–30:51) |
| Distal small intestine | 01:13 (00:51–01:42) | 06:59 (02:13–11:08) | 13:36 (09:01–26:29) | 21:49 (14:02–35:03) |
| Proximal colon | 01:18 (00:49–01:55) | 01:25 (00:14–02:54) | 15:49 (03:20–25:55) | 16:37 (06:44–21:54) |

Values are presented as arithmetic mean (range), n = 5 per group.

## Supplementary Fig. S1. cAMP accumulation assays demonstrating in vitro potency of peptides (J229 and MEDI7219) in CHO cell lines expressing human GLP-1R with (a) 0.1% BSA or (b) 4.4% HSA or in (c) human EndoC-βH1 cell lines, compared with reference peptide semaglutide.


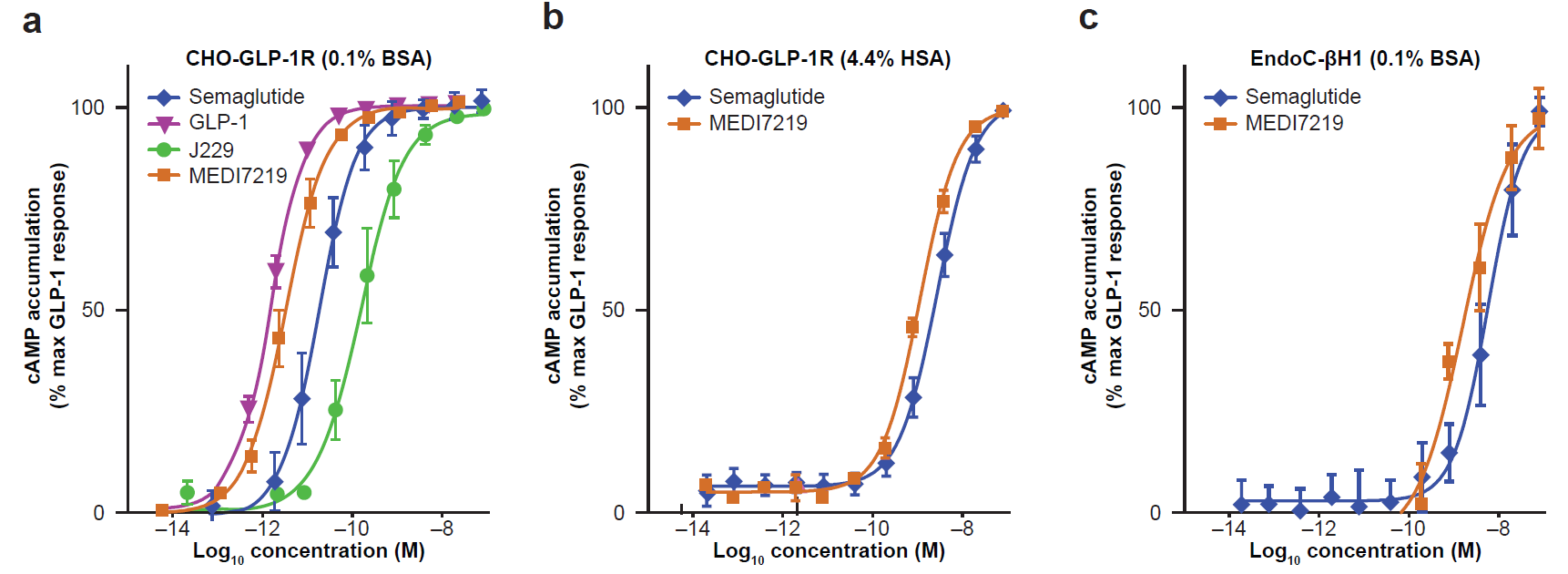


## Human pharmacokinetic/pharmacodynamic modeling

The systemic pharmacokinetic (PK) parameters estimated in pooled intravenous dog PK analysis were allometrically scaled to humans, with a clearance of 9.54 L/day and a volume of distribution of 8.12 L. Absorption parameters, absorption rate constant (Ka) and bioavailability are assumed to be the same between dog and human. Ka is 0.554/hour as estimated from the equation:

$$T_{max}=\frac{1}{\left( Ka-Kel \right)}*ln\frac{Ka}{Kel}$$

where T_max_ (time at which maximal concentration is observed after oral dosing) is 4 hours and Kel (elimination rate constant) is 0.0848/hour in dogs. Human bioavailability is estimated to be 4%.

These parameters were used to simulate human PK profiles for the proposed oral and intravenous doses summarized in Supplementary Fig. S1.

## Supplementary Fig. S2. Simulated MEDI7219 concentrations in humans for oral and intravenous routes of administration.
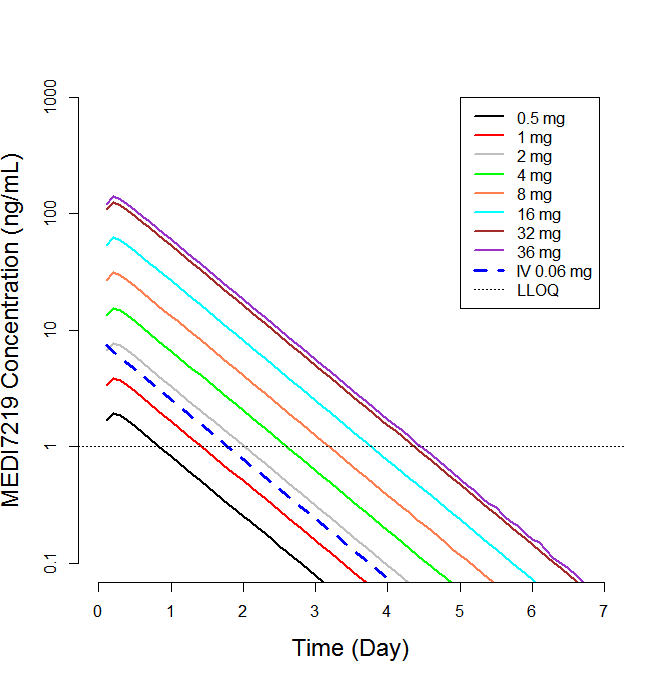


## Supplementary Fig. S3. Liquid chromatography mass spectrometry (LC/MS) spectra of J211, J229 and MEDI7219. (a) LC of J211, (b) MS of J211, (c) LC of J229, (d) MS of J229, (e) LC of MEDI7219 and (f) MS of MEDI7219.

**a
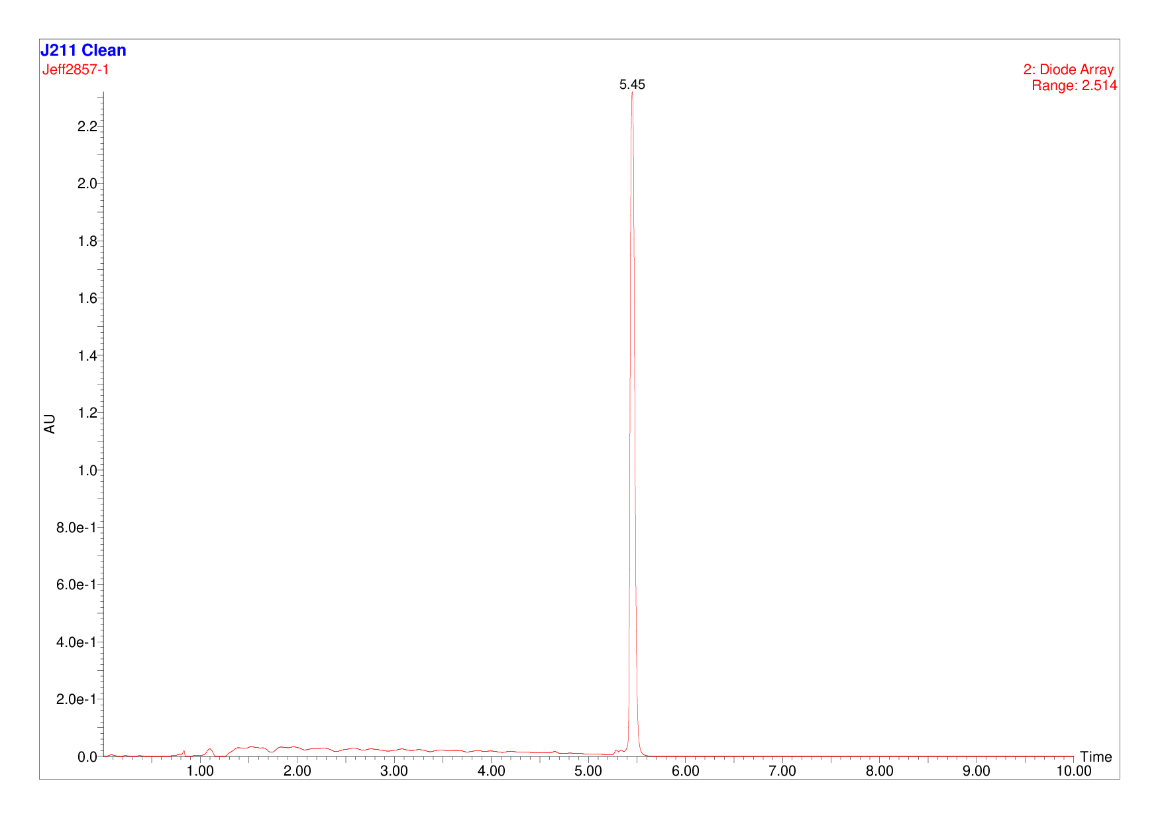
**

**b**
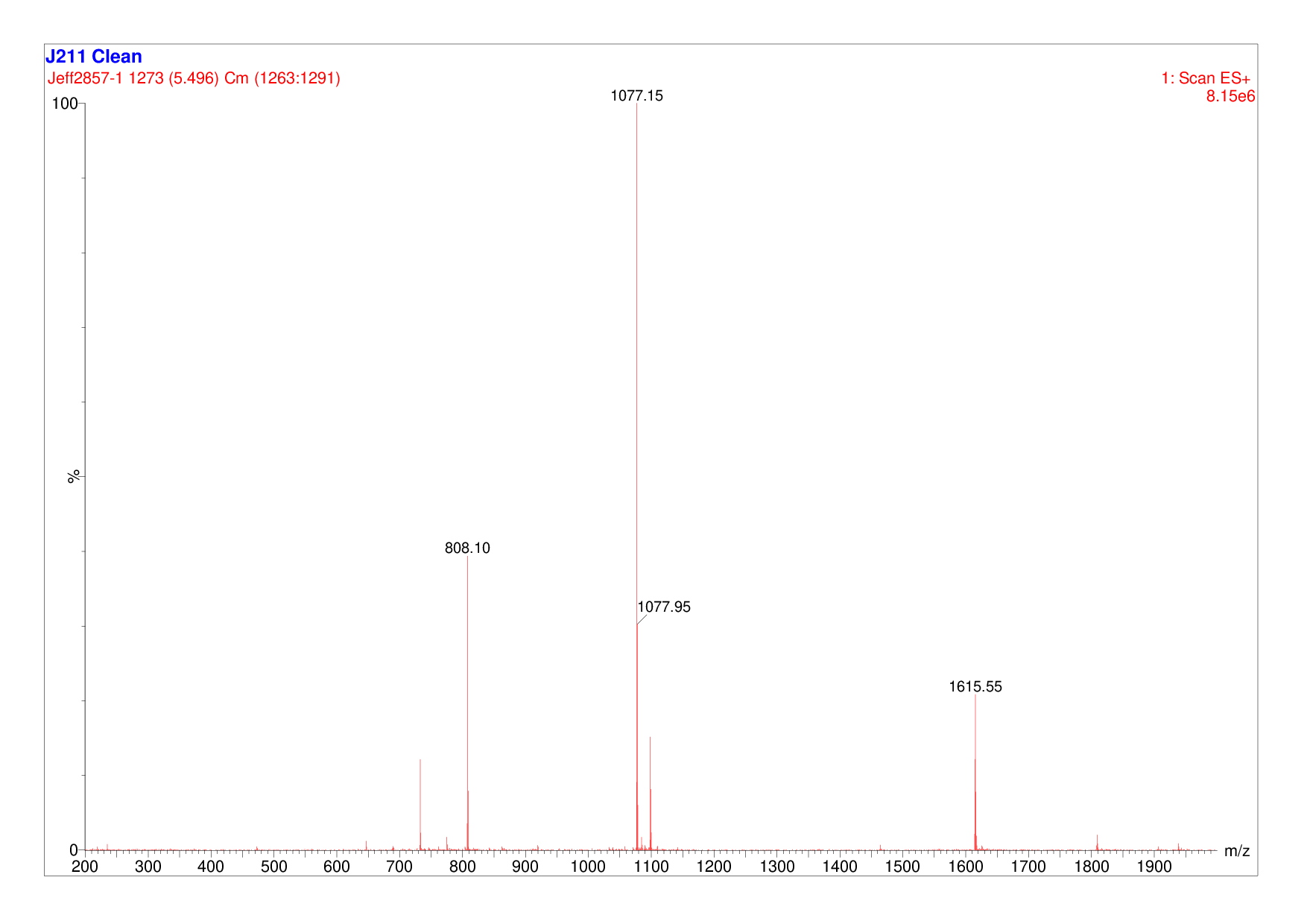


**c**
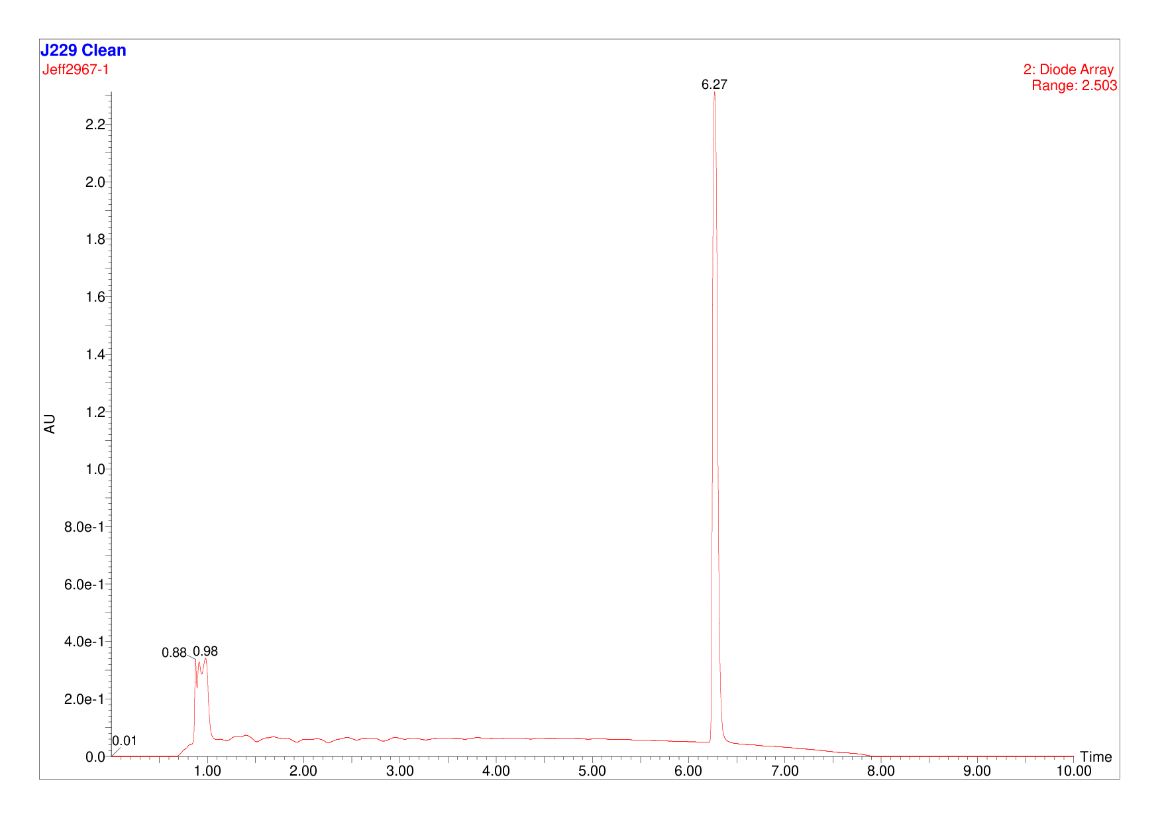


**d**
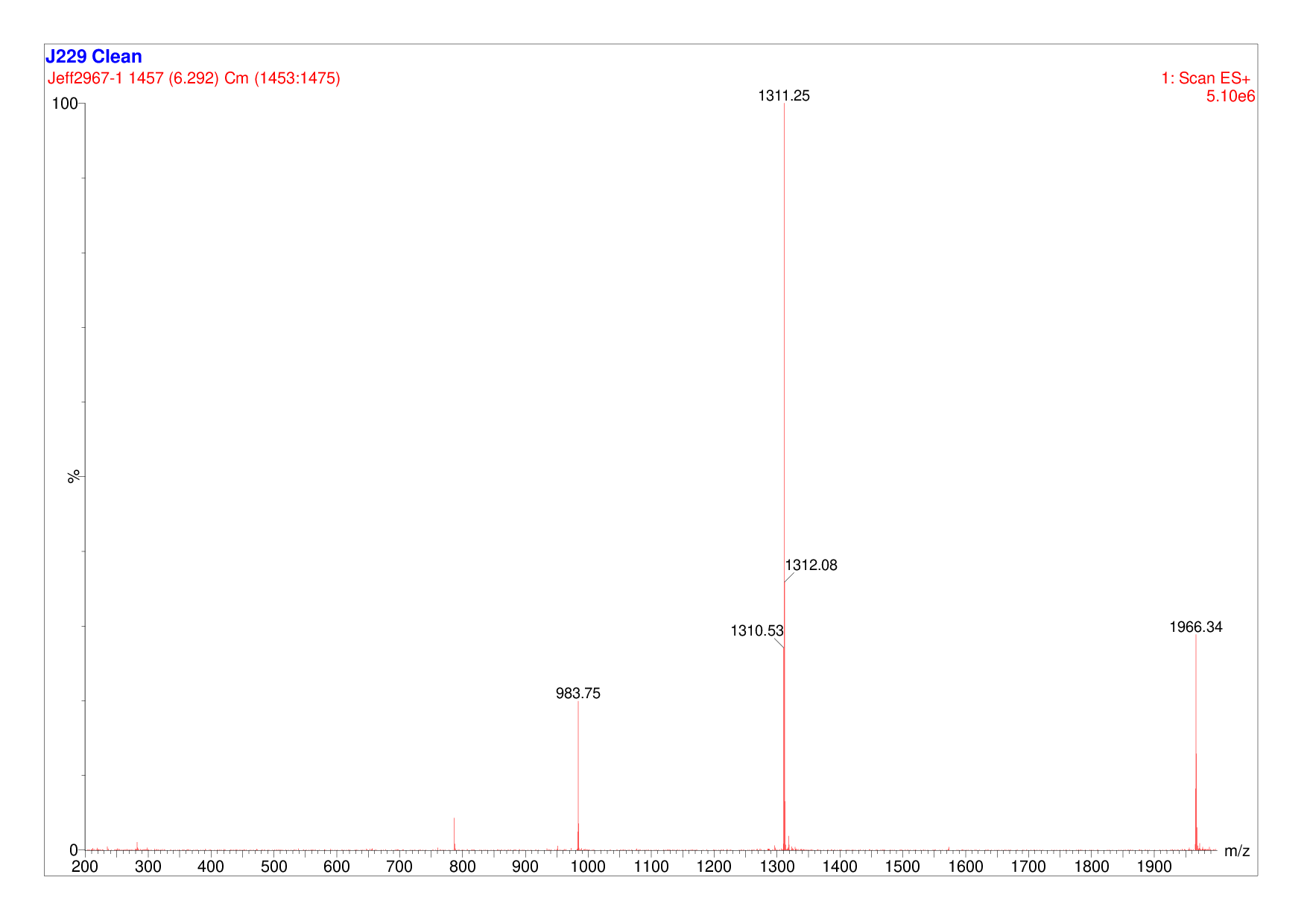


**e**
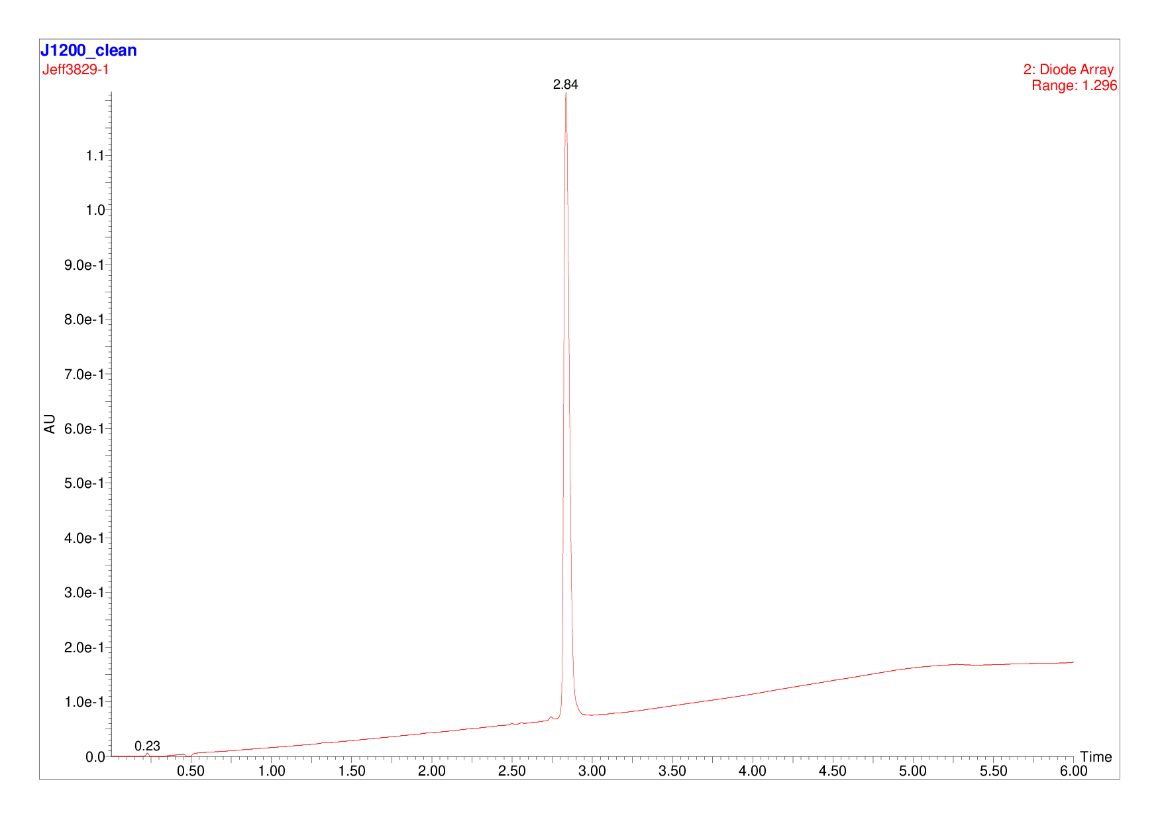


**f**
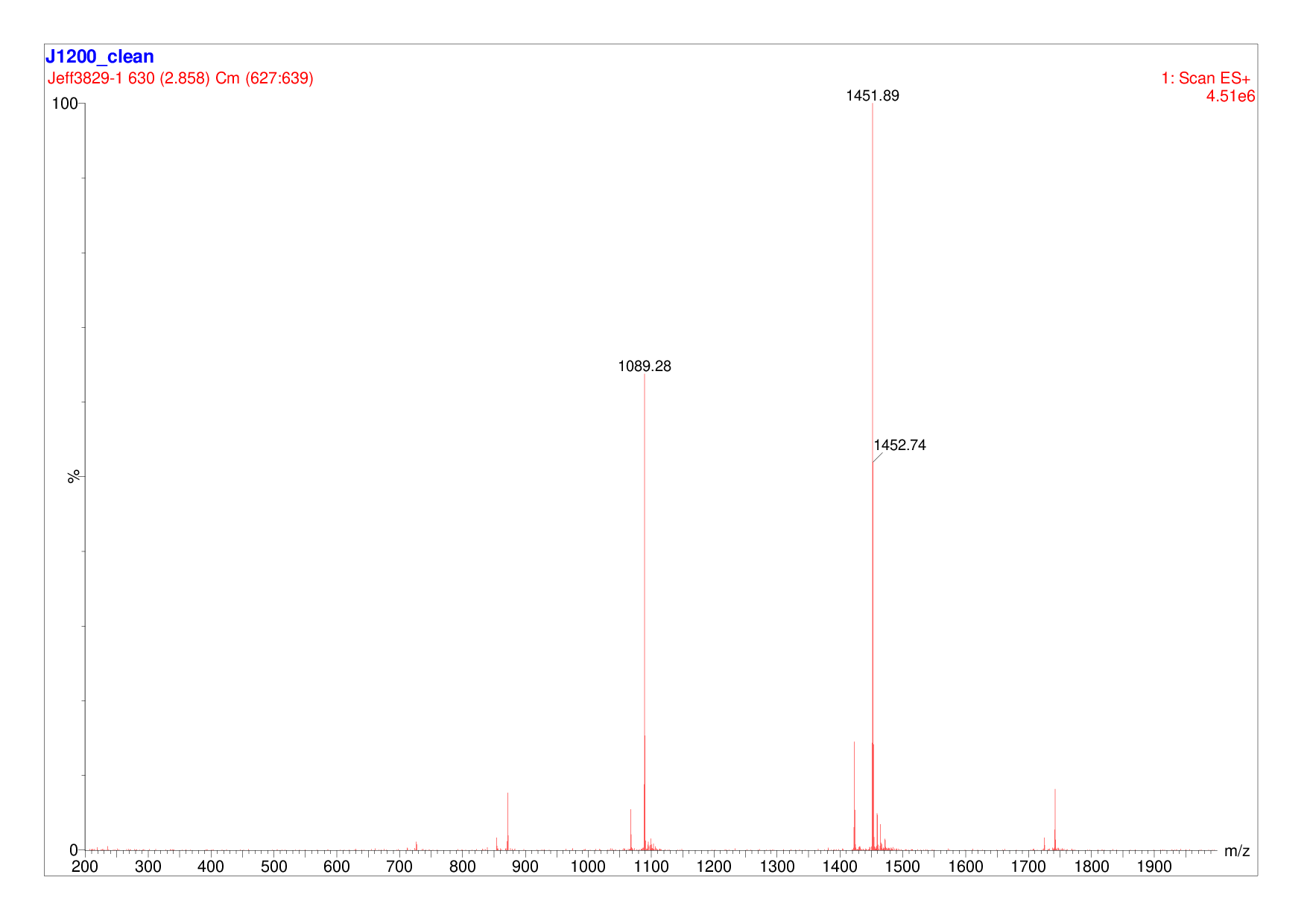

Supplement: Supplementary file 1 — Supplementary Information. [file 41598_2021_1750_MOESM1_ESM.docx]
